# Mental Health among Migrants in Shenzhen, China: Does it Matter Whether the Migrant Population is Identified by Hukou or Birthplace?

**DOI:** 10.3390/ijerph15122671

**Published:** 2018-11-27

**Authors:** Min Yang, Martin Dijst, Marco Helbich

**Affiliations:** 1 Department of Human Geography and Spatial Planning, Faculty of Geosciences, Utrecht University, 3584 CB Utrecht, The Netherlands; m.helbich@uu.nl; 2 Luxembourg Institute of Socio-Economic Research (LISER), 4366 Luxembourg City, Luxembourg; martin.dijst@liser.lu

**Keywords:** migration, mental health, hukou, birthplace, China

## Abstract

Massive rural–urban migration in China has drawn attention to the prevalence of mental health problems among migrants. Research on the mental health of Chinese migrants has a narrow focus on rural–urban migrants, emphasizing the institutional role of hukou in migrant mental health. We argue that the heterogeneity of migrants, including their place of origin and whether they are temporary or permanent migrants, should be taken into account when trying to understand the meaning of migration as an actual movement from one place to another. The data used for this study is from a cross-sectional survey (*N* = 855) conducted in Shenzhen to compare the differences in migrants’ mental health that arise when using the two definitions (e.g., hukou and birthplace). Binary logistic regression models were estimated to assess the associations between people’s mental health and migration, while controlling for settlement experiences, self-reported physical health, and sociodemographics. The results reveal inconsistent findings across both definitions: general migrants by birthplace were found to be unlikely to have mental problems compared to non-migrants, whereas temporary migrants were at higher risk of mental problems. The study provides important evidence that different migrant groups have different mental health outcomes. The choice of the definition used influences both migrant group selection and the actual linkage between migration and mental health.

## 1. Introduction

Since the 1980s, China has undergone rapid urbanization accompanied by massive migration from the countryside to urban areas. Approximately 286.5 million migrants left their rural homes to seek better life opportunities in cities in 2017 [1].

Increasing population mobility triggered interest among academics in migrants’ physical and mental health in host cities [2,3,4]. Yet previous studies of Chinese migrants’ mental health have been producing mixed findings [5]. Some suggested migrants’ mental health status was worse than urban non-migrants because of limited social welfare, low socioeconomic status, acculturation issues, risks of discrimination, and marginalization [4,6,7]. Others observed the healthy migrant phenomenon where migrants reported better health status than non-migrants [8]. Some suggested that since the process of migration is difficult and stressful, people with relatively better physical and mental health are more likely to migrate while people with poorer health are more likely to remain home [9]. However, the initial health advantages of migrants may reduce over time with increasing length of residence in host society [10]. In addition, the observed healthy migrant phenomenon could be caused by a selection bias where those migrants who are physically and mentally less healthy have returned home for treatments. Thus, they are excluded from the sample selection in host cities [11]. In short, migrants’ mental health status can be influenced by various factors that may cause positive or negative mental health outcomes.

In Chinese migration studies, hukou—China’s household registration system—is seen as an institutional barrier preventing migrants from enjoying equal rights in host cities, for example, the right to access housing, employment, education, and social and healthcare services [12]. Consequently, most studies on migrant mental health in China focus on rural-to-urban migrants (*Nongmin Gong*) who hold rural hukous but work and reside in urban areas [13,14]. In addition to a disadvantaged socioeconomic status caused by hukou situation [2], rural–urban migrants were found to be more likely to experience discrimination and stigmatization in host cities [15,16]. Social and spatial exclusion was also reported by rural–urban migrants [5,17,18]. Rural–urban migrants’ self-identification and sense of belonging are constantly challenged in host cities, leading to a higher risk of depression and other mental health problems [19,20]. Meanwhile, migrants were forced to live in certain parts of the city, primarily in suburban areas and “urban villages” that have poor housing conditions due to housing disadvantages created by the hukou system [18,21]. The lack of a local hukou was clearly shown to increase a migrant’s living stress and mental health problems.

However, mental health studies in China have a narrow focus on rural–urban migrants, neglecting the heterogeneity of migrants and excluding a significant portion of the migrant population. First, migrants are different based on the temporary or permanent nature of migration, which is partly indicated by hukou registration [22]. “Temporary migrants” are people whose place of residence is different from their place of registration. In contrast, “permanent migrants” are people who transferred their place of registration to their current place of residence [23,24]. Many migrants expect to settle down in their host cities for a long time or even permanently [25]. Therefore, transferring their hukous to the host cities has become the ultimate goal of migrants [26]. The current hukou regulation allows individuals to transfer their hukou to a host city by pursuing higher education, working in the public sector, or having family connections [27,28]. It can also be done by purchasing an urban dwelling, although it takes years of hard work to save enough to purchase an urban dwelling [26]. Permanent migrants are ignored in most studies since they are no longer registered as “migrants” in the census once they have obtained a local hukou [23]. Second, migrants are different in terms of their place of origin, namely rural–urban migrants and urban–urban migrants. Yet, urban–urban migrants are not included in migrant mental health studies. Compared to rural–urban migrants, urban–urban migrants may experience less urban–rural differences and therefore adapt to their new lives much quicker. In addition, they are more determined to establish their homes permanently in host cities and to obtain local hukous [29,30]. Besides, the initial health status may differ between migrants who came from rural areas and urban areas, due to the big difference between the physical and natural environment in rural and in urban settings [31]. Some reported that rural residents are in general healthier than urban residents because they have a better natural environment, a less stressful and unhealthy lifestyle, and healthier diets [31,32,33].

To address the narrow focus on rural–urban migrants, some attempts at broadening the population of migrants have been made. Skilled migrants have received attention from researchers with respect to issues of housing conditions, residential mobility, and social integration [29,30]. In migrant health studies, the healthy migrant phenomenon was examined among both rural–urban migrants and urban–urban migrants, where place of birth was used to differentiate between migrants and non-migrants [11]. Despite these efforts, little progress has been made with respect to the heterogeneity of migrants and identifying migrant mental health associations for a broader migrant population. Since the institutional disadvantage due to the absence of local hukou only applies to temporary migrants who migrate without hukou transfers, we would expect different mental health performance between temporary migrants and the general migrant population.

To sum up, this paper addresses the research gap caused by the overlooked heterogeneity of migrants in existing migrant mental health studies. The ignorance of the greater migrant population, including their place of origin and whether they are temporary or permanent migrants, might be problematic and biased when trying to identify the mental health effects of migration. To address the gap, we analyzed novel data from a cross-sectional survey conducted in Shenzhen to investigate the differences that arise when exploring migration–mental health associations for: (1) temporary migrants, including both rural–urban and urban–urban migrants, without local hukous; and (2) the general migrant population, which includes all people who experienced migration regardless of their hukou status, but using birthplace as criterion of migration.

## 2. Materials and Methods

### 2.1. Study Area

The city of Shenzhen has been the prime destination for migrants since the start of China’s economic reform in the 1980s. By the end of 2015, Shenzhen had a population of 11.37 million people, of whom only 3.54 million have local hukous, which means that over 70% of Shenzhen’s population are temporary migrants. This figure would be even larger if migrants were defined by their birthplace instead of possession of a Shenzhen hukou. Being a special economic zone as well as the financial and logistic center of southern China, the city is home to both labor-intensive and high-tech manufacturing industries, as well as modern service industries. The diversity of the economic structure has attracted a broad array of migrants, ranging from low-skilled to high-skilled and highly educated migrants. All this made Shenzhen an ideal city for this research.

### 2.2. Research Design and Sampling

A cross-sectional survey investigating the mental health of migrants in Shenzhen was conducted between January and April 2017. A multistage sampling procedure was used to select respondents. The two inner city districts of Nanshan and Futian, and the two suburban districts of Baoan and Longgang, were selected as sampling areas. Within each area, five neighborhoods were identified based on various neighborhood characteristics, including work unit compound, inner-city village, open-market apartment community, social housing, and factory dormitory. However, minor deviation from the initial sampling design was necessary because a few of the neighborhoods were gated and a security pass was required to gain admission. We therefore also considered the surrounding neighborhoods within the selected district that match the abovementioned neighborhood characteristics. Next, housing units in the identified neighborhoods were randomly selected without replacement to avoid repeated measurements. The head of each household was asked to fill out the questionnaire. If the household head was not available, another household member older than 18 was asked to complete the questionnaire. Since college students were considered a special group of migrants in terms of residence type (concentrated in school-provided dormitories), they were excluded from the sampling. In total, 855 questionnaires were considered valid for the analysis. This overall sample size fits the rule of thumb that the minimum sample size should be 15 times the number of independent variables [34].

This study was approved by the Ethics Committee of the Faculty of Social and Behavioral Sciences of Utrecht University and filed under number FETC17-132.

### 2.3. Data

*Mental health*: The dependent variable for the analysis of mental health status was derived from the General Health Questionnaire (GHQ-12), which contains 12 items measuring potential mental health problems [35]. The validity and reliability of the GHQ-12 questionnaire have been extensively tested in previous empirical studies [36,37,38]. For this study, the well-tested Chinese version was utilized [39,40]. The GHQ-12 questionnaire contains six questions measuring positive attributes (e.g., being able to concentrate, feeling useful, and enjoying life), and six that measure negative attributes (e.g., loss of sleep, losing confidence, and feeling unhappy). The total score is obtained by summing up the scores on each individual question, and ranges from 0 to 12. In this paper, we use the binary scoring method for case identification: 0 refers to an absence of mental health problems, and 1 refers to possible mental health deficits [35]. Cronbach’s α of the GHQ-12 was 0.85 for our sample.

*Migration*: Migrants were defined by two variables. The first is based on the conventional hukou definition of temporary migrants: respondents who reported having Shenzhen hukous were identified as non-migrants, and people without Shenzhen hukous were identified as migrants. The second variable was birthplace which is used to identify migrants in general terms: people born in Shenzhen were identified as non-migrants, and people born outside Shenzhen were identified as migrants.

In addition, three variables representing people’s settlement experience in Shenzhen were considered. First, information about the length of residence in Shenzhen was collected. The more time that people spend in a host city, the more likely they are to better adjust themselves to the new society. Length of residence is generally associated with people’s positive feelings and interactions with the place [41]. Second, we asked the respondents about house ownership. Owning a dwelling in a host city contributes to migrants’ psychological wellbeing and enhances their connections to that city [42,43]. Third, residential mobility was measured by the number of residential moves within Shenzhen. Studies suggest that people who are residentially stable, report better general mental health compared to those who are residentially highly mobile [44]. Migrants in China have substantially higher mobility rates and more residential instability compared to local urban residents [28,45].

*Control variables*: The following confounding variables were also considered: age in years, gender, level of education, personal monthly income in RMB (RMB 1 = USD 0.15), and number of jobs held. Since people’s mental health is related to their physical health [46,47], we adjusted for that on the basis of a self-rated question on a Likert scale of 1 to 5. This type of operationalization is widespread [48,49] and is well validated for the Chinese context [9].

### 2.4. Statistical Analysis

Descriptive analyses were conducted to reveal the properties of each variable. We utilized χ^2^ tests for categorical variables and independent *t*-tests for continuous variables to investigate differences concerning the prevalence of mental health problems between migrants and non-migrants, as well as across the two definitions of migration.

Since the dependent variable was binary coded, we estimated binary logistic regression models to test the associations between the prevalence of mental health problems and the independent variables. The following four models were fitted into two groups: Model 1 tested the relationship between migration and mental health using migrants defined according to hukou. Model 2 tested the same relationship in Model 1, adjusting for confounders including settlement experience in Shenzhen, self-reported physical health, and sociodemographic attributes. In Model 3, we tested the relationship between migration and mental health using birthplace to define migrants. Model 4 tested the same relationship as in Model 3, adjusting for the confounders. Variables with *p* < 0.05 were considered statistically significant. SPSS 24 (IBM, New York, NY, USA) was used for the statistical analyses.

## 3. Results

The 855 participants had a GHQ-12 mean score of 1.36 (standard deviation, SD = 1.637). The cut-off point for unlikely/likely to have mental health problems was set at 1/2, which is in line with previous studies [50]. The prevalence of mental health problems (GHQ-12 score ≥ 2) was 28.8% for the sample.

Table 1 presents the descriptive statistics. The sample comprised 457 (53.5%) temporary migrants according to hukou definition. The general migrant group comprised 591 people (69.1%) when using birthplace as the definition.

The results of the χ^2^ tests as shown in Table 1, show that significantly higher mental health problem prevalence rates were present among temporary migrants (defined by hukou), non-migrants (defined by birthplace), people who did not own their dwellings, people who reported “fair and poor” general physical health, and people whose personal monthly incomes were less than RMB 4000. The *t*-test shows a significantly lower average number of years of residence in Shenzhen and a higher average number of residential moves among the group that had a high prevalence of mental problems. Variance inflation factors provided no evidence for multicollinearity.

Table 2 presents the results of the binary logistic regression models. Consistent with the χ^2^ test, both definitions of migrants were found to be significantly associated with the prevalence of mental health problems independently. Migrants defined by hukou type were at higher risk of having mental health problems, whereas lower risks were found when migrants were defined by birthplace. The relationships remained significant after controlling for confounding factors. The effects of the control variables were different when different definitions were applied. When migrants were defined according to hukou, length of residence in Shenzhen was negatively correlated with prevalence of mental health problems. The association was no longer significant when migrants were defined according to birthplace. Instead, gender was found to be significantly associated with the prevalence of mental health problems. A significant positive relationship was found between residential mobility and prevalence of mental health problems in both models. This means that making multiple housing moves in Shenzhen might be a risk factor for mental health problems. The results also show that the worse people perceive their physical health condition, the more likely they are to have mental health problems. Finally, people with monthly incomes of RMB 4001–8000 are significantly less likely to have mental health problems compared to those who earn less than RMB 4000 per month. In order to test whether the covariables are different across migrants and non-migrants, the models were re-run with interaction terms as well. As the interactions were insignificant, the results are not reported.

## 4. Discussion

This study addressed the nature of migration and its relation to mental health by comparing two groups of migrants, namely temporary migrants (according to their current hukou registrations) and the general migrant group by birthplace (with hukou transfers). The results show that a significant number of migrants had managed to transfer their hukous to Shenzhen (134 cases, 15.6% of the total sample). We found a prevalence rate of mental health problems of 28.8% of the sample, assessed by GHQ-12 with a cut-off score of 2. Using this measurement, the prevalence rate of temporary migrants was 30.6% and 26.6% for non-migrants, while the number became 26.4% for general migrants and 34.1% for non-migrants when birthplace was used to identify migrants. The regression analysis showed migration to be significantly associated with the prevalence of mental health problems. Yet, the directions of the association differ when defining migrants differently. Temporary migrants identified by hukou were significantly less mentally healthy than non-migrants while the general migrants by birthplace are mentally healthier than non-migrants. As a large proportion of migrants managed transferring their hukous to Shenzhen after arrival, we conclude that great caution is needed in defining migrants when investigating the relationship between migration and mental health in China.

The choice between hukou and birthplace is not merely a selection of sample size. Instead, it has implications on understanding migrants’ mental health status in the Chinese context. Our results showed that temporary migrants (non-Shenzhen hukou) have a significantly higher chance in suffering from mental health problems comparing to non-migrants in Shenzhen (Shenzhen hukou). This finding is in line with previous empirical findings [3,4]. The hukou system is a key factor for health inequality among Chinese migrants and the biggest institutional barrier for migrants’ desired urban lives [51,52]. Hukou has been closely linked with healthcare services, including medical insurance, access to professional health consultation, and access to proper medication [5,6]. The inequality issue caused due to the lack of local hukous also appears in employment, social security, and housing. For instance, migrants have limited access to subsidized housing in host cities and the majority of them end up in poor living conditions. Another major issue for migrants is the difficulty of acquiring a place in school in host cities for their children [51]. As a result, migrants have to leave their children in their home towns for schooling and suffer from family separation, which challenges both migrants and their left-behind children’s mental health [53].

The general migrants, on the other hand, contains a broader selection of migrant types, including those who transferred their hukous to Shenzhen [28]. Reference [51] reported that transferring hukous to host cities significantly improves migrants’ mental wellbeing, since doing so addresses most of the inequality issues caused by the hukou system. In addition, hukou migrants are more likely to have better employment opportunities, higher incomes, and a higher level of education [51]. This financial and human capital contributes to better mental health [54,55]. Moreover, international migration studies observed the healthy migrant phenomenon where immigrants reported better health performance than the native-born when they arrived in destination countries [3]. We found a similar effect for general migrants by birthplace. The intertwined relationship between migration and mental health is well represented by the healthy migrant phenomenon where healthier people tend to be migrants and move further away from home [9], resulting in a better initial health status than non-migrants. Yet, migrants’ mental health status may vary during their migration experiences, including being away from home and their original social network, as well as coping and adapting experiences in destinations. Moreover, migration is associated with improved socioeconomic status and quality of life. Apart from economic gains, migrants also benefit from relatively good social resources, such as better schooling for their children and improved living conditions, both of which seem to enhance mental health status [56].

As evidenced by our study, the choice between two definitions of migrants has significant implications. The hukou-based definition selects migrants whose place of residence differs from their hukou registration, and in most cases, these are rural–urban migrants [23]. This definition is suitable for specific rural–urban migrant studies that address urban and rural areas [3,14] and focus on inequality between migrants and non-migrants in Chinese cities [6,13,15]. The birthplace definition, however, includes more people who changed their residence during any of their life stages. We believe that birthplace represents migration experiences more accurately, as it allows the incorporation of changes in exposure due to residential changes.

In addition to the different associations between mental health and migration for different migrant groups, the results also showed that residential mobility in Shenzhen plays a significant role in migrants’ mental health status. We found that frequent residential moves were associated with a higher prevalence of mental health problems, which is congruent with previous studies [43,44]. Empirical studies have reported a higher residential mobility rate in migrants compared to local people [45,57]. In comparison to the locals, migrants tend to start at the bottom of the housing market due to their limited social and economic resources. They improve their living conditions over time, whereas locals have accumulated both social and economic resources over generations, and therefore enter owner-occupied housing earlier [28]. Our findings also suggest that self-reported physical health is strongly associated with mental health where the better physical health reported, the less chance people have mental health problems. This result is in line with existing empirical results [3]. In addition, a higher income was found to contribute to better mental health performance from our analysis. This finding is consistent with earlier research [3,54]. All these factors mentioned above showed the same direction of association with prevalence of mental health problems, regardless which migrant group was analyzed. It means that these factors, including residential mobility rate in Shenzhen, perceived physical health, and income are crucial factors affecting people’s mental health performance.

There are several policy implications based on our findings. First, migrants who lack urban hukous are the most vulnerable group in terms of mental health problems. Policy makers should reduce the gap between local hukou and non-local hukou to better facilitate migrants’ lives in cities. Second, although migrants with local hukou seem to have a lower risk of having mental health problems, pronounced residential mobility might act as an additional risk factor. Efforts should be made to make migrants feel more at home, for example, by granting them access to the public renting system to improve the quality of their initial housing. Social support should be provided at both the community and the neighborhood level to facilitate migrants’ connection to the local environment and social groups, thereby enhancing their residential experiences and reducing their tendency to move.

Several limitations should be considered when interpreting the results. First, we did not include circulating migrants, namely migrants who constantly move back and forth between their original homes and the city, or migrants who had moved back to their original homes. Since these groups of migrants may differ in terms of sociodemographic attributes, they may hold different expectations of their migrant life compared to our sample. Second, the sample was recruited only in Shenzhen, which is a migration hotspot. This might restrict the generalization of our findings to other Chinese cities. Third, due to the cross-sectional nature of our study, causal relationships between the prevalence of mental health problems and migration-related factors cannot be inferred. Further longitudinal studies are required to establish and verify causation between mental health and migration. Finally, we cannot rule out endogeneity issues, because covariables could be correlated with the error term. Future studies should address this issue by means of instrumental variables [58].

Despite these limitations, several strengths need to be highlighted. First, the study represents an initial effort to investigate the impact of different definitions of migrants in analyzing the relationship between migration and mental health. It draws attention to the potential impacts on analytical outcomes for different migrant groups. Second, the study stresses the importance of broadening the research scope of migrants in China according to birthplace. Without its role of identifying migrants, hukou still serves as an important factor that indicates people’s resettlement status and is closely associated with people’s mental health. Therefore, the research represents an important exploration of how migration is related to mental health in urban China.

## 5. Conclusions

Migrant mental health research in China has been limited by the narrowed selection of rural–urban migrants. Yet, no knowledge has been built to understand the difference between the two definitions of migrants, namely hukou and birth place. By comparing the migrant populations identified according to each of the two definitions and their roles in analytical models, we found that the definition of migrant had a significant influence on the association between mental health and migration. Moreover, there are different implications behind each definition, including migrants’ socioeconomic status and, more importantly, their capacity and intention to stay permanently in host cities. We suggest that future research could build on our outcomes by taking a closer look at the meanings represented by the two definitions. Studies could include migrant heterogeneity by looking at migrants’ birthplace, so that we gain a better understanding of how and why certain migrants become better off than others. Our findings also suggest that along with migration, residential mobility, physical health, and personal income play significant roles in the prevalence of mental health problems.

## Figures and Tables

**Table 1:** Descriptive statistics and mental health prevalence rate.

Variable	Category	Whole Sample	Prevalence of Mental Health Problems	
		*N* (%)/Mean	*N* (%)/Mean	χ^2^ Test/*t*-Test
**Definition of migrants**				
Temporary migrants by hukou	Non-migrants	398 (46.5%)	106 (26.6%)	1.66 *
	Migrants	457 (53.5%)	140 (30.6%)	
General migrants by birthplace	Non-migrants	264 (30.9%)	90 (34.1%)	5.27 *
	Migrants	591 (69.1%)	156 (26.4%)	
**Settlement experience**				
Length of residence in Shenzhen (years)		25.5	23.8	2.83 *
Housing ownership	No	586 (68.5%)	184 (31.4%)	6.27 *
	Yes	269 (31.5%)	62 (23.0%)	
Residential mobility in Shenzhen		1.7	2.0	−2.87 **
**Physical health**				
Self-reported physical health	Fair and poor	295 (34.5%)	138 (46.8%)	72.08 **
	Good	246 (28.8%)	52 (21.1%)	
	Very good	190 (22.2%)	35 (18.4%)	
	Excellent	124 (14.5%)	21 (16.9%)	
**Sociodemographic variables**				
Age (years)		31.5	30.4	1.74
Gender	Female	392 (45.8%)	109 (27.8%)	0.33
	Male	463 (54.2%)	137 (29.6%)	
Education	High school or lower	235(27.5%)	78 (33.2%)	3.09
	Bachelor’s or lower	569 (66.6%)	154 (27.1%)	
	Master’s or above	51 (5.9%)	14 (27.5%)	
Personal income	<RMB 4000	199 (23.2%)	76 (38.2%)	11.72 **
	RMB 4001–8000	351 (41.1%)	95 (27.1%)	
	>RMB 8000	305 (35.7%)	75 (24.6%)	
Number of jobs	None	57 (6.7%)	20 (35.1%)	1.37
	One job	664 (77.6%)	186 (28.0%)	
	>One job	134 (15.7%)	40 (29.9%)	

* *p* < 0.05; ** *p* < 0.01.

**Table 2:** Results of the binary logistic regressions (*N* = 855).

Independent Variables	Model 1		Model 2		Model 3		Model 4	
	Coef.	Stand. Err.	Coef.	Stand. Err.	Coef.	Stand. Err.	Coef.	Stand. Err.
**Temporary migrants by hukou (reference: Non-migrants)**								
Migrants	0.430 **	0.154	0.517 *	0.206				
**General migrants by Birthplace (reference: Non-migrants)**								
Migrants					−0.366 *	0.160	−0.630 **	0.203
**Settlement experience**								
Length of residence in Shenzhen (years)			−0.029 *	0.013			−0.001	0.013
Housing ownership (reference: No)								
Housing ownership (Yes)			0.023	0.211			−0.367	0.202
Residential mobility			0.137 **	0.047			0.130 **	0.047
**Perceived physical health (reference: Poor and fair)**								
Good			−1.201 **	0.201			−1.193 **	0.201
Very good			−1.333 **	0.229			−1.358 **	0.230
Excellent			−1.590 **	0.283			−1.660 **	0.285
**Sociodemographic variables**								
Gender (Female)			0.298	0.171			0.354 *	0.170
Age (years)			0.011	0.015			−0.011	0.015
Education (reference: High school and lower)								
Bachelor’s degree			−0.072	0.201			−0.265	0.198
Master’s degree			0.266	0.397			0.034	0.389
Personal monthly income (reference: <RMB 4000)								
RMB 4001–8000			−0.487 *	0.217			−0.470 *	0.217
>RMB 8000			−0.407	0.255			−0.420	0.255
No. of jobs (reference: None)								
One job			−0.145	0.325			−0.061	0.328
>One job			0.025	0.374			0.075	0.377
**Constant**	−1.146 **	0.117	0.012	0.522	−0.659 **	0.130	0.910	0.511
**Nagelkerke R^2^**	0.013		0.164		0.019		0.169	

* *p* < 0.05; ** *p* < 0.01.
